# Supplementary material for: Towards a Long-Read Sequencing Approach for the Molecular Diagnosis of RPGRORF15 Genetic Variants
Source: Int J Mol Sci. 2023 Nov 28;24(23):16881. doi: 10.3390/ijms242316881 (PMC10706286; doi:10.3390/ijms242316881)
Supplement: Supplementary file 1 [file ijms-24-16881-s001.zip › ijms-2695240-supplementary.pdf]

International Journal of
Molecular Sciences

MDPI

Supplementary Material

Table S1. NGS sequencing coverage of RPGR orf15 exon in the analyzed samples. When a specific coverage was not identified in any patient, "/" was reported as Mean exone taget hole.

Coverage range	Mean exone target hole (bp)	Number of patients
47.5-50.0 %	899	1
50.0-52.5 %	845	5
52.5-55.0 %	800	8
55.0-57.5 %	754	19
57.5-60.0 %	709	22
60.0-62.5 %	669	12
62.5-65.0 %	623	7
65.0-67.5 %	599	1
67.5-70.0 %	/	0
70.0-72.5 %	/	0
72.5-75.0 %	/	0
75.0-77.5 %	/	0
77.5-80.0 %	/	0
80.0-82.5 %	329	2

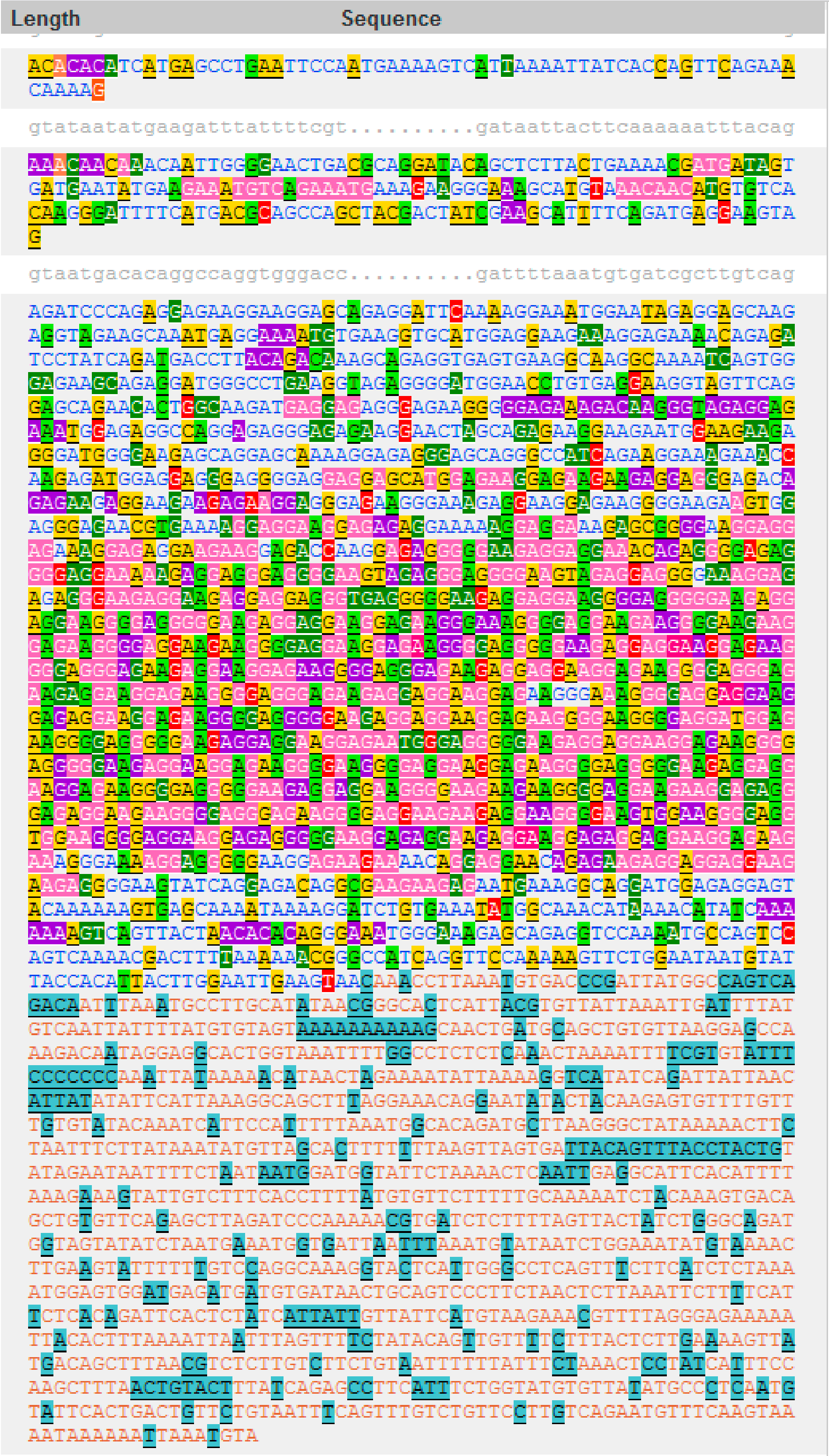

Figure S1. RPGRORF15 exon 15 sequence on Ensembl database. Colored residues represent residues in which genetic variants are found (ORF15 is a mutation hotspot of RPGR). Light blue: 3 prime UTR variants; Green: Coding sequence variants; Violet: Frameshift variants; Pink: Inframe deletions variants; Yellow: Missense variants; Dark Pink: Protein altering variants; Orange: Splice donor variants; Light orange: Splice region variants; Red: Stop gained variants; Light green: Synonymous variants.

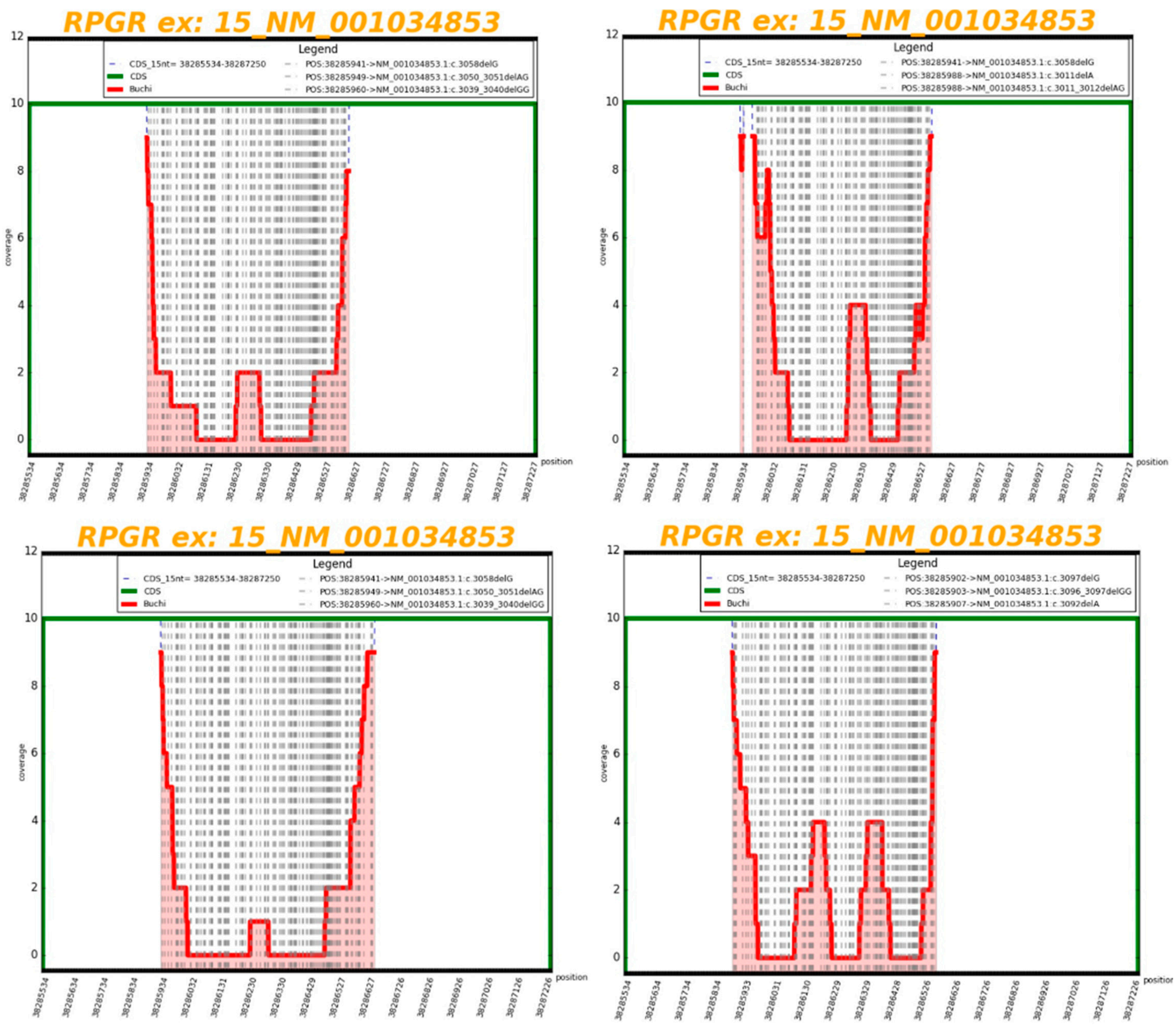

Figure S2. Examples of NGS sequencing coverage pitfalls in RPGRORF15 exon 15. Average coverage of RPGR is 60X, while orf15 region has a coverage lower than 10X. X axes represent nucleotide position, while Y axes represent coverage.

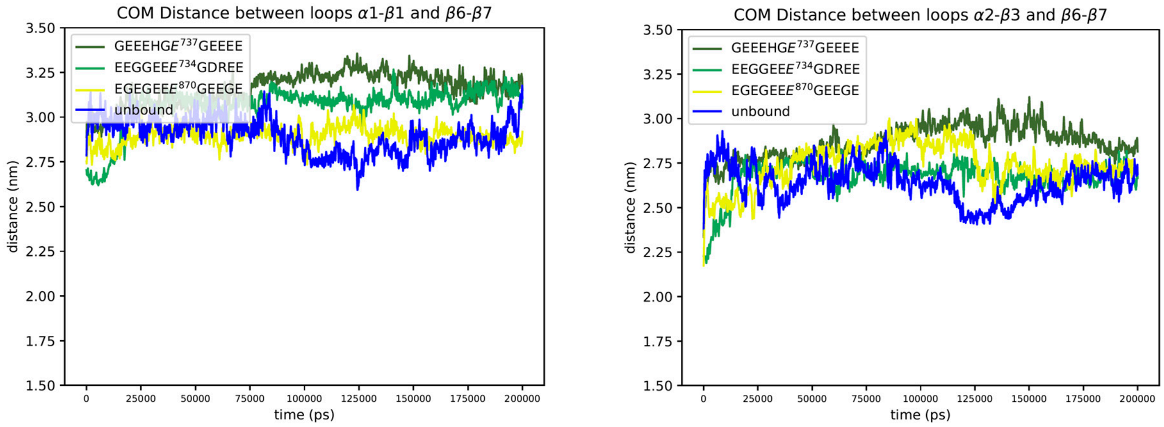

Figure S3. COM distance between the respective loops forming the binding cavity. Both the distances conform to a narrow stance in the free (unbound) TTLL5 as compared to the bound complex.
